# Antimicrobial Compounds Produced by Vaginal *Lactobacillus crispatus* Are Able to Strongly Inhibit *Candida albicans* Growth, Hyphal Formation and Regulate Virulence-related Gene Expressions

**DOI:** 10.3389/fmicb.2017.00564

**Published:** 2017-04-04

**Authors:** Shuai Wang, Qiangyi Wang, Ence Yang, Ling Yan, Tong Li, Hui Zhuang

**Affiliations:** Department of Microbiology and Center of Infectious Disease, School of Basic Medical Sciences, Peking University Health Science CenterBeijing, China

**Keywords:** *Lactobacillus*, *Candida albicans*, microbiota, vulvovaginal candidiasis, antimicrobial activity, yeast-tohyphae

## Abstract

The female vaginal environment contains diverse microorganisms, and their interactions play significant roles in health and disease. *Lactobacillus* species are the predominant vaginal microorganisms in healthy women and relevant as a barrier to defense against pathogens, including *Candida albicans*. The yeast-to-hyphae transition is believed to be a determinant of *C. albicans* pathogenesis. In this study, we investigated the effects of vaginal isolates of *L. crispatus* (seven strains), *L. gasseri* (six strains), and *L. jensenii* (five strains) on growth, hyphal formation and virulence-related genes expression of *C. albicans* ATCC 10231. We found that the *L. crispatus* showed the most significant antimicrobial activities in microplate-based liquid medium assay (*P* < 0.05). All seven cell-free supernatants (CFS) from *L. crispatus* strains reduced the growth of *C. albicans* by >60%. The effects might be due to their productions of some secretory antimicrobial compounds in addition to H_2_O_2_ and organic acids. Furthermore, each of the CFS of *Lactobacillus* strains was found to significantly suppress the yeast-to-hyphae transition of *C. albicans* under hyphae-inducing conditions (RPMI 1640 medium supplemented with 10% fetal bovine serum). The hyphae inhibition rates of *C. albicans* treated by CFS from *L. crispatus*, *L. gasseri*, and *L. jensenii* were 88.3 ± 3.02%, 84.9 ± 6.0%, and 81.9 ± 6.2%, respectively. Moreover, the expression of hyphae-specific genes (*ALS3*, *HWP1*, *ECE1*, *EAP1*, and *SAP5*) and transcriptional regulatory genes (*EFG1*, *TEC1*, and *NRG1*) were analyzed using quantitative real-time PCR. The results demonstrated that *L. crispatus* CFS significantly down-regulated the expression of hyphae-specific genes *ALS3* (0.140-fold)), *HWP1* (0.075-fold), and *ECE1* (0.045-fold), while up-regulated the expression of the negative transcriptional regulator gene *NRG1* with 1.911-fold. The antimicrobial compounds from *L. crispatus* B145 against *Candida* growth were heat stable and protease resistance, but those against hyphal formation were partially sensitive to the same treatments. Our novel findings suggest that *L. crispatus*, a dominant *Lactobacillus* species associated with a healthy vagina, could strongly inhibit *C. albicans* growth and hyphal formation. *L. crispatus* might repress the expression of hyphae-specific genes (*ALS3*, *HWP1*, and *ECE1*) in a *NRG1*-dependent manner. Besides, *L. crispatus* B145 is highly worthwhile for probiotic investigation.

## Introduction

*Candida albicans* is an opportunistic pathogen and the most prevalent fungal species of the human microbiota (da Silva Dantas et al., 2016). In individuals with healthy immune systems, *C. albicans* asymptomatically colonizes mucosal surfaces. However, in some conditions this species can cause infections, such as VVC (Nobile and Johnson, 2015). The overgrowth and morphological transition (e.g., yeast-to-hyphae transition) of *C. albicans* are very important determinants to promote the conversion from a commensal to a pathogen (Jacobsen et al., 2012). VVC is responsible for a great morbidity among women of reproductive-age and significant burden to the health care system due to rising vaginitis-related health care costs. It is estimated that about 75% of all women at the childbearing age are afflicted by VVC at least once in their lifetime with ∼40–50% experiencing at least one additional episode of infection, ∼5–8% of women suffer from at least four recurrent VVC per year (Peters et al., 2014; Cassone, 2015).

It is well accepted that the interactions between *C. albicans* and other components of the resident bacteria, particularly *Lactobacillus* spp., play important roles in determining the commensal or pathogenic outcome of this fungus (Förster et al., 2016). Lactobacilli are predominant microorganisms in the normal vaginal microbiota, where the most frequently isolated species are *L. crispatus*, *L. gasseri*, and *L. jensenii* (Petrova et al., 2015). In a healthy status, *C. albicans* and lactobacilli naturally co-inhabit in the female genitourinary tract, where the former lives as a minority co-habitant. It is reported that lactobacilli could inhibit the overgrowth of *C. albicans* by producing a wide variety of secondary metabolites with antimicrobial activity, such as lactic acid, hydrogen peroxide (H_2_O_2_), bacteriocin-like compounds, and biosurfactants (Borges et al., 2014). For instance, bacteriocin L23 produced by *L. fermentum* isolate displayed inhibition effect on *C. albicans* growth (Pascual et al., 2008). However, the mechanisms underlying anti-*Candida* activity are still not clearly understood.

When the balance of vaginal flora is disturbed by the weakened immune system, antibiotic usage and other risk factors, the lactobacilli biomass would decrease and its species composition might shift, while *C. albicans* could overgrow and undergo a morphogenetic change from a round-ovoid typical yeast cell to a hyphal growing organism (Ma et al., 2012; Cassone, 2015). The yeast-to-hyphae transition is believed to be a determinant of *C. albicans* pathogenesis, and it could be triggered by various environmental cues *in vitro*, including neutral pH, presence of serum or *N*-acetyl glucosamine, elevated carbon dioxide concentration and physiological temperature (Mayer et al., 2013; Palková and Váchová, 2016). In addition, the morphological transition from yeast to hyphae is regulated by a complex network of signaling pathway in *C. albicans*. Several virulence-related genes that are specifically expressed in hyphae have been suggested to play essential roles in *C. albicans* pathogenesis (Sudbery, 2011; Jacobsen et al., 2012). For example, the hyphal wall protein 1 gene (*HWP1*), agglutinin-like protein gene 3 (*ALS3*), and extent of cell elongation gene 1 (*ECE1*) encode cell surface proteins, which are important for hyphal growth and adhesion to host cells (Fan et al., 2013; Vila et al., 2016). Expressions of these hyphae-specific genes are positively regulated by transcription factors Efg1 and Tec1, and are also negatively regulated by the transcriptional repressors Nrg1 (Shapiro et al., 2011).

The use of probiotic lactobacilli against VVC has emerged as an attractive therapeutic option in the era of antibiotic resistance (Matsubara et al., 2016a). Several clinical trials reported that the commercially available probiotic *Lactobacillus* spp., such as *L. rhamnosus* GR-1, *L. reuteri* RC-15, and *L. plantarum* I1001, are beneficial to women’s urogenital health by decreasing the risk of VVC (Reid et al., 2003; Martinez et al., 2009; Murina et al., 2014; Palacios et al., 2016). However, they are seldom reported as the main resident *Lactobacillus* species in healthy vagina. Moreover, little is known about the roles and underlying mechanisms of yeast-to-hyphae transition in *C. albicans* regulated by lactobacilli, especially by those resident *Lactobacillus* species (Orsi et al., 2014b; Matsubara et al., 2016b; Oliveira et al., 2016).

The aim of this study was to investigate the effects of the most frequently found *Lactobacillus* spp. in the healthy vagina, i.e., *L. crispatus*, *L. gasseri*, and *L. jensenii*, on growth, hyphal formation and virulence-related genes expression of *C. albicans*. We found that all 18 *Lactobacillus* strains tested could inhibit the growth and hyphal formation of *C. albicans* in a strain-specific manner. In addition, for the first time, we demonstrated that *L. crispatus* inhibited *C. albicans* hyphal formation by down-regulating the hyphae-specific genes *ALS3*, *HWP1*, and *ECE1* possibly through up-regulating the expression of transcriptional repressor gene *NRG1*. Having the strongest inhibition effects on *C. albicans* growth and hyphal formation, and being the most commonly found resident *Lactobacillus* species in the healthy vagina, *L. crispatus* shows a great potential as a probiotic candidate.

## Materials and Methods

### Strains and Culture Conditions

Eighteen *Lactobacillus* strains of three different species (*L. crispatus*, *L. gasseri*, and *L. jensenii*) were originally isolated from human vaginal secretion (Wang et al., 2014, 2015). *L. acidophilus* ATCC 4356 and *C. albicans* ATCC 10231 were purchased from the China General Microbiological Culture Collection Center.

All *Lactobacillus* strains were routinely grown in MRS medium (BD, Sparks, MD, USA) and incubated in an anaerobic atmosphere (BD, Sparks, MD, USA) at 37°C for 48 h. *C. albicans* was routinely grown for 12 h at 37°C in YPD medium.

### Determination of H_2_O_2_ Production

The qualitative plate assay for H_2_O_2_ production was carried out according to the method of Xu et al. (2008). In brief, following 48 h of anaerobic incubation on the TMB-HRP-MRS plates [MRS agar containing 250 μg/mL 3, 3′, 5, 5′-tetramethylbenzidine (TMB; Amresco, Solon, OH, USA) and 50 μg/mL horseradish peroxidase (HRP; Biodee, Beijing, China)], the colonies of H_2_O_2_-producing strains turned blue after 30 min air exposure. Based on the color intensity, the potential of H_2_O_2_ production could be divided into four categories: negative (-), weakly positive (+), positive (++), and strongly positive (+++) (Xu et al., 2008). *L. acidophilus* ATCC 4356 was used as a positive control, and graded as a strong H_2_O_2_ producer.

### Preparation of Cell-Free Supernatant (CFS) from *Lactobacillus* Strains and pH Measurement

Lactobacilli were grown in MRS broth (pH 6.5) for 48 h at 37°C in anaerobic condition. CFS was prepared by centrifuging the culture at 8000 rpm for 15 min at 4°C and then filtered through 0.22 μm filters (Millipore, Bedford, MA, USA). The pH value of CFS was measured by using a pH meter (PHS-3E, INESA Scientific Instrument, Shanghai, China). CFS was stored as single-use aliquots at -20°C until needed.

### Antimicrobial Activity of Lactobacilli against *C. albicans*

The modified agar overlay method was used to determine the antimicrobial activity of lactobacilli against *C. albicans* (do Carmo et al., 2016). Ten microliter of *Lactobacillus* spp. suspensions (∼10^6^ CFU/mL) were spotted onto MRS agar plates, and incubated anaerobically for 48 h. The MRS agar plates containing the growth of lactobacilli (≈8 mm in diameter) were overlayed with a *C. albicans* suspension (∼10^7^ CFU/mL) in 10 mL of YPD soft agar (0.5% agar). The inhibitory effect of MRS was tested as a negative control on each plate. After aerobic incubation for 24 h, inhibition zones were read. The GIA was calculated as follows: GIA (mm) = (IZD – CD)/2, where IZD was inhibition zone diameter and CD was colony diameter of a *Lactobacillus* spot. A GIA grading system was applied, in which GIA < 0.5 mm was recorded as negative (–), (0.5, 2) mm as weak positive (+), (2, 3.5) mm as intermediate positive (++), and ≥ 3.5 mm as strong positive (+++).

### Antimicrobial Activity of CFS against *C. albicans*

A microplate assay was used to evaluate the antimicrobial activity of the CFS against *C. albicans* according to Parolin et al. (2015) with some modifications. A mixture of 100 μL *C. albicans* suspensions in YPD broth (∼10^6^ CFU/mL) and 100 μL tested CFS was added to each well of a sterile 96-well microplate (Costar 3799, Corning, NY, USA). MRS broth was used instead of CFS as a positive growth control. The plate was incubated at 37°C aerobically for 24 h, and the growth of *C. albicans* was recorded as OD at 630 nm wavelength using a microplate reader (Model 680, Bio-Rad, Hercules, CA, USA). The growth inhibition rate (%) was expressed as: (OD_control_ – OD_CFS_)/OD_control_ × 100.

### Effect of CFS on *C. albicans* Hyphal Formation

Hyphal growth assay of *C. albicans* was performed in RPMI 1640 medium supplemented with 10% FBS (Sun et al., 2015). We used both solid and liquid medium assays to evaluate the effects of CFS on *C. albicans* hyphal formation. In these two assays, MRS broth instead of CFS was used as control. All assays were repeated for five times.

For solid medium assay, *C. albicans* cells from an overnight culture were washed with PBS and spread (∼100 colonies per plate) on plates of solidified medium supplemented with 50 μL of CFS. Plates were aerobically incubated at 37°C for 5 days. Images of colony edges were obtained using a stereomicroscope (SZ66, Optec, Chongqing, China).

To assess the effect of CFS in liquid medium, a mixture of 900 μL *C. albicans* suspensions (∼10^6^ CFU/mL) in medium and 100 μL CFS was added into each well of a 48-well microplate, and aerobically incubated at 37°C for 4 h. Quantification of inhibition of the yeast-to-hyphae transition was accomplished by counting the number of individual yeast cells *versus* the number of hyphae in the population under a light microscope (AxioCam MRc5; Carl Zeiss, Jena, Germany). More than 100 cells were counted for each well in duplicate, and all assays were repeated for five times. The hyphae inhibition rate (%) was calculated as: (Hyphae%_control_ – Hyphae%_CFS_)/Hyphae%_control_ × 100.

### Quantitative Real-Time Reverse Transcription PCR (qRT-PCR)

To determine the effects of CFS in RPMI 1640 + 10% FBS broth on the transcription of *C. albicans* genes related to yeast-to-hyphae transition, gene expression levels of *GSP1*, *NRG1*, *TEC1*, *EFG1*, *EAP1*, *SAP5, ALS3*, *HWP1*, and *ECE1* were evaluated by two-step qRT-PCR (Sun et al., 2015; James et al., 2016). Total RNA of *C. albicans* was extracted using a Yeast RiboPure RNA Purification kit (CoWin Biosciences, Beijing, China). Concentration, purity, and quality of the isolated RNA samples were determined using a Nano Drop One Spectrophotometer (Thermo Scientific, Waltham, MA, USA). RNA (1 μg) from each sample was immediately reverse transcribed into cDNA using a Prime Script RT reagent kit (Takara, Tokyo, Japan) according to the manufacturer’s instructions. The conditions for reverse transcription were 15 min at 37°C, 5 s at 85°C. qRT-PCR was performed with 80 ng cDNA template and SYBR Premix Ex Taq (Takara) in ABI StepOne Real Time PCR System (Applied Biosystems). Primer sequences used for amplification of specific genes were shown in **Table 1**. *GSP1*, which was not transcriptionally regulated in the morphogenesis switch, served as the internal control (Sun et al., 2015). The following parameters were used for qRT-PCR: one cycle at 95°C for 15 s, 40 cycles at 95°C for 5 s and then 60°C for 30 s. Specificity of the primers was confirmed by melting curve analysis. The generated *C*_T_ values of target genes were normalized to the *C*_T_ value of reference gene *GSP1*. Relative expression fold changes were evaluated by ΔΔ*C*_T_ method using 2^-ΔΔC_T_^ formula (Livak and Schmittgen, 2001).

**Table 1:** List of primers used for qRT-PCR experiments.

Gene	Function	Primer sequence (5′–3′)
*GSP1*	GTP-binding protein (Reference gene)	F: TGAAGTCCATCCATTAGGAT


		R: ATCTCTATGCCAGTTTGGAA
*NRG1*	Negative transcriptional regulator	F: CACCTCACTTGCAACCCC


		R: GCCCTGGAGATGGTCTGA
*TEC1*	Conserved filamentation activator	F: AGGTTCCCTGGTTTAAGTG


		R: ACTGGTATGTGTGGGTGAT
*EFG1*	Filamentous growth protein	F: TATGCCCCAGCAAACAACTG


		R: TTGTTGTCCTGCTGTCTGTC
*EAP1*	Extracellular adherence protein	F: CTGCTCACTCAACTTCAATTGTCG


		R: GAACACATCCACCTTCGGGA
*SAP5*	Secreted aspartyl proteinase	F: CAGAATTTCCCGTCGATGAGA


		R: CATTGTGCAAAGTAACTGCAACAG
*ALS3*	Agglutinin-like protein	F: CTAATGCTGCTACGTATAATT
		R: CCTGAAATTGACATGTAGCA
*HWP1*	Hyphal cell wall protein	F: TGGTGCTATTACTATTCCGG
		R: CAATAATAGCAGCACCGAAG
*ECE1*	Extent of cell elongation protein	F: GCTGGTATCATTGCTGATAT


		R: TTCGATGGATTGTTGAACAC

### Statistical Analysis

All assays were carried out in triplicate on at least three different occasions with independently grown cultures unless otherwise stated. All statistical analysis was performed using IBM SPSS Statistics 20.0 software program (IBM, Armonk, NY, USA). Statistical comparison between two groups was performed by Student’s *t*-test. Comparisons among multiple groups were performed by one-way ANOVA followed by LSD test. A *P*-value < 0.05 was considered statistically significant.

## Results

### Antimicrobial Activity and H_2_O_2_ Producing Ability of Lactobacilli

The antimicrobial activity of lactobacilli against *C. albicans* was evaluated using agar overlay method. The results showed that 15 of 18 (83.3%) *Lactobacillus* strains isolated from vaginal mucosa had inhibition activities on *C. albicans* growth (**Table 2**), among which more than half (8/15, 53.3%) had high inhibition activities ranked as +++.

**Table 2:** Characteristics of 18 vaginal *Lactobacillus* strains.

Strain	pH of CFS	H_2_O_2_ producing ability^a^	GIA^b^
*L. crispatus*			
A014	3.91	++	+++
A055	3.95	++	++
B125	3.93	+++	++
B135	3.94	+++	+++
B145	3.93	+++	+++
B422	3.93	++	+++
B535	3.93	+++	++
*L. gasseri*			
A054	3.92	-	++
B062	3.86	+	+
B351	3.93	+++	+++
B451	3.95	-	++
B542	3.97	-	++
B554	3.93	-	+++
*L. jensenii*			
A083	4.09	+++	-
B021	4.09	+++	-
B101	4.02	+++	+++
B161	3.96	++	-
B511	4.16	+++	+++

^a^*The H_2_O_2_ producing ability of *Lactobacillus* strains was graded as strongly positive (+++), positive (++), weakly positive (+), and negative (-) according the color intensity of colony on the TMB-MRS plate. ^b^The GIA was calculated by subtracting a CD of a *Lactobacillus* spot from the IZD and expressed as follows: GIA = (IZD – CD)/2. GIA < 0.5 mm was recorded as negative (-), [0.5, 2) mm as weak positive (+), [2, 3.5) mm as intermediate positive (++), and ≥ 3.5 mm as strong positive (+++)*.

The production of H_2_O_2_ by *Lactobacillus* strains was tested using the semi-qualitative TMB-HRP-MRS assay (**Table 2**). The strong-, moderate-, weak- and non-producers were 50.0% (9/18), 22.2% (4/18), 5.6% (1/18), and 22.2% (4/18), respectively.

### Effect of CFS on *C. albicans* Growth

**FIGURE 1:**
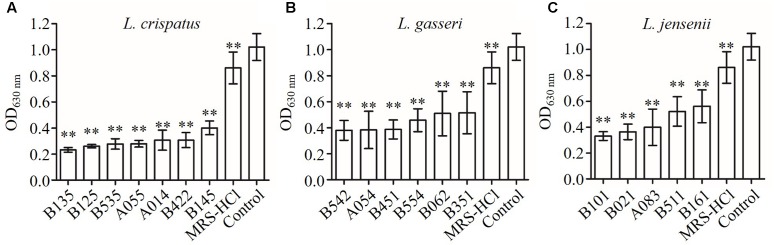
**Inhibition effects of CFS from different *Lactobacillus* strains on *Candida albicans* growth in YPD broth at 24 h after inoculation. (A)** *L. crispatus*, **(B)** *L. gasseri*, **(C)** *L. jensenii*. Each bar is the mean ± SD from three independent experiments. ^∗∗^*P* < 0.01 compared to control by Student’s *t*-test.

The strains of lactobacilli were assayed for their ability to produce inhibition compounds in CFS against the growth of *C. albicans*. The average pH value of CFS was 4.0 (in the range of 3.8–4.2), which was lower than fresh MRS broth (initial pH 6.5) (**Table 2**). To investigate whether low pH contributed to the growth inhibition effects of CFS, fresh MRS broth was adjusted to pH 4.0 with HCl (MRS-HCl). In the presence of CFS or MRS-HCl, the growth of *C. albicans* was significantly reduced when compared to the MRS control in general (**Figure 1**) (*P* < 0.01). In addition, while the MRS-HCl could significantly inhibit the growth of *C. albicans* (OD_630nm_ MRS-HCl vs. control: 0.861 ± 0.122 vs. 1.021 ± 0.103, *P* = 0.008), each of the CFS could further inhibit *C. albicans* growth with significant difference compared to MRS-HCl (*P* < 0.01).

**FIGURE 2:**
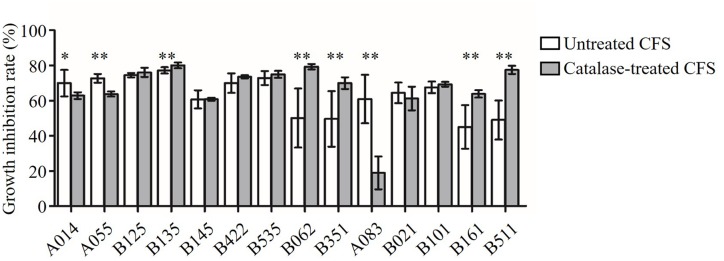
**Inhibition of *C. albicans* growth by CFS and catalase-treated CFS**. Growth inhibition rate (%) = (OD_control_ – OD_CFS_)/OD_control_ × 100. The statistical comparison between the catalase untreated and treated groups for each of the CFS was performed by Student’s *t*-test. ^∗^*P* < 0.05, ^∗∗^*P* < 0.01.

In order to test whether the antimicrobial activity was due to H_2_O_2_, the CFS was treated with catalase enzyme (1 mg/mL; Sigma, St. Louis, MO, USA) at 37°C for 1 h before approaching to the inhibition assay (**Figure 2**). The antimicrobial activity of six CFS (B125, B145, B422, B535, B021, and B101) was resistance to the catalase treatment (*P* > 0.05). These results suggested that these six strains might produce anti-*Candida* compounds with strong activities beyond the effect of H_2_O_2_. After catalase treatment, the growth inhibition rates of three CFS (A014, A055, and A083) were found to decrease, indicating that the observed anti-*Candida* effects of these three strains were partially attributed to the production of H_2_O_2_ (*P* < 0.05). However, it was strange that the growth inhibition abilities of five CFS (B135, B062, B351, B161, and B511) were found to significantly increase after catalase treatment.

### Effect of CFS on *C. albicans* Hyphal Formation

**FIGURE 3:**
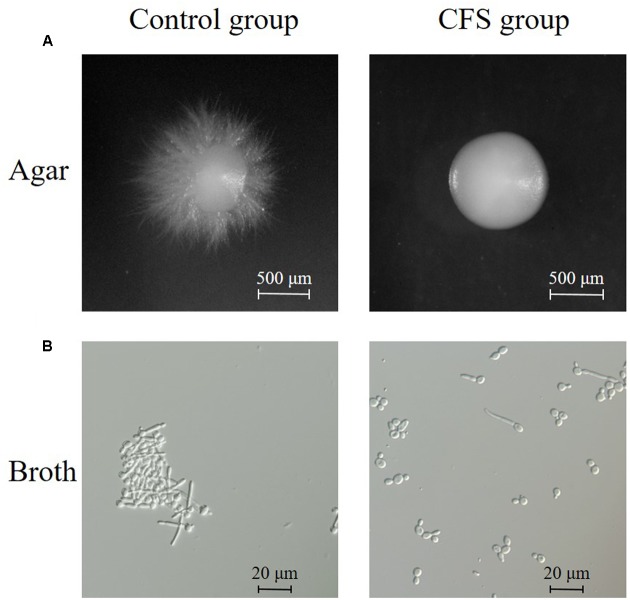
**Inhibition of hyphal formation by CFS. (A)** Effect of CFS on *C. albicans* hyphal formation on solid RPMI 1640 + 10% FBS medium. Plates were incubated at 37°C for 5 days. Images of colony edges were obtained using a stereomicroscope, original magnification: 6.6×. **(B)** Effect of CFS on *C. albicans* hyphal formation in liquid RPMI 1640 + 10% FBS medium for 4 h at 37°C. Images of cellular morphology were obtained using a light microscopy, original magnification: 400×.

**FIGURE 4:**
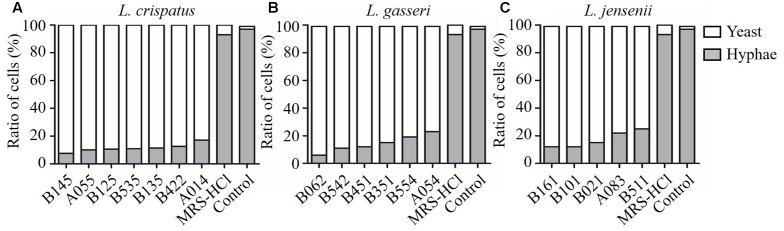
**Inhibition effects of CFS from different *Lactobacillus* strains on *C. albicans* hyphal formation. (A)** *L. crispatus*, **(B)** *L. gasseri*, **(C)** *L. jensenii*. Effect of the addition of CFS on the yeast-to-hyphae conversion induced by liquid RPMI 1640 + 10% FBS medium at 37°C for 4 h in microtiter wells.

The effect of CFS on the hyphal formation was evaluated in RPMI 1640 agar medium containing 10% FBS (hypha-inducing condition). The yeast form and filamentous-proned colonies of *C. albicans* were counted. The results showed that the treatment with CFS was able to inhibit filamentation. **Figure 3A** was an example of *C. albicans* colony at absence or presence of CFS. In addition, the effect of CFS on the hyphal formation was evaluated in liquid medium supplemented with CFS. The results showed that most *C. albicans* cells grew as yeast (**Figure 3B**). As seen in **Figure 4**, about 98% of untreated cells formed hyphae and pseudohyphae over 4 h time course. When treated by MRS-HCl, there was a significant lower ratio of hyphal cells compared to that treated by MRS control (92.6 ± 8.0% vs. 97.8 ± 4.6%, *P* = 0.038). However, each of the CFS showed more significant inhibition effects on *C. albicans* hyphal formation compared to that of MRS-HCl (*P* < 0.01).

### Comparison of Inhibitory Activities of *Lactobacillus* Species

**FIGURE 5:**
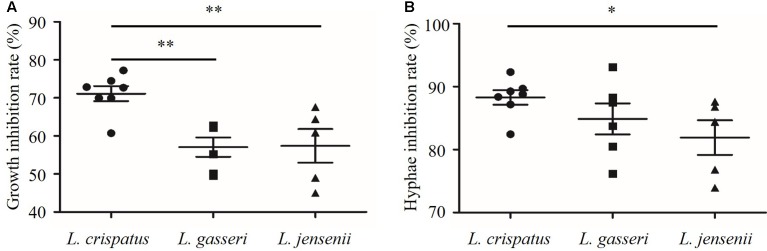
**Inhibition of growth and hyphal formation of *C. albicans* by CFS from *L. crispatus*, *L. gasseri*, and *L. jensenii*. (A)** Growth inhibition rate (%) = (OD_control_ – OD_CFS_)/OD_control_ × 100, **(B)** Hyphae inhibition rate (%) = (Hyphae%_control_ – Hyphae%_CFS_)/Hyphae%_control_ × 100. Comparisons among three groups were performed by one-way ANOVA followed by LSD test. ^∗^*P* < 0.05, ^∗∗^*P* < 0.01.

As shown in **Figure 5**, the growth and hyphal formation of *C. albicans* were inhibited by CFS from *L. crispatus*, *L. gasseri*, and *L. jensenii*. The growth inhibition rate of *C. albicans* treated by *L. crispatus* (71.1 ± 5.2%) was significantly higher than those treated by *L. gasseri* (57.1 ± 6.3 %) or *L. jensenii* (57.4 ± 9.9%), respectively (*P* < 0.01). The hyphae inhibition rate of *C. albicans* treated by *L. crispatus* (88.3 ± 3.02%) was significantly higher than that by *L. jensenii* (81.9 ± 6.2%) (*P* < 0.05). However, there was no significant difference between *L. crispatus* and *L. gasseri* treatments (88.3 ± 3.0% vs. 84.9 ± 6.0%, *P* > 0.05). Taking into consideration of growth and hyphal formation inhibition activities, we chose *L. crispatus* CFS to study the effects on the transcription of *C. albicans* virulence-related genes.

### Modulation of *C. albicans* Gene Expression by CFS

**FIGURE 6:**
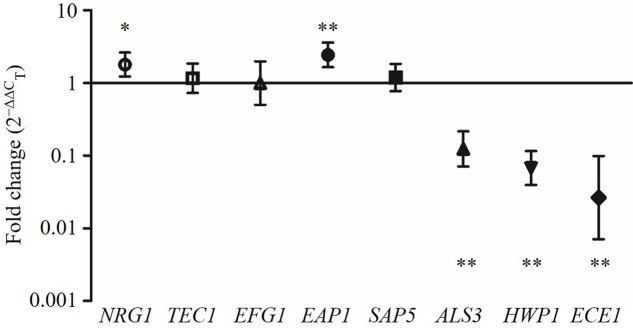
**The expression levels (fold change) of virulence-related genes in *C. albicans* treated with CFS**. ^∗^*P* < 0.05 and ^∗∗^*P* < 0.01 compared to control by Student’s *t*-test.

The expression levels of the hyphae-specific genes (*ALS3*, *HWP1*, *ECE1*, *EAP1*, and *SAP5*) and transcriptional regulatory genes (*EFG1*, *TEC1*, and *NRG1*) were quantified in *C. albicans* incubated with CFS by qRT-PCR. **Figure 6** showed that CFS significantly down-regulated the expression of the hyphae-specific genes *ALS3* (0.140-fold), *HWP1* (0.075-fold), and *ECE1* (0.045-fold), the differences were statistically significant compared to MRS control (*P* < 0.01). The gene expression level of *SAP5* (1.283-fold) had no statistical difference compared to the control (*P* > 0.05). Strikingly, *EAP1* gene, which involved in adhesion and biofilm formation, was found to be significantly up-regulated by CFS treatment (2.590-fold, *P* < 0.01). In addition, the expression level of negative transcriptional regulator *NRG1* was also found to be significantly up-regulated (1.911-fold, *P* < 0.05), while two hyphae activator regulatory genes *TEC1* (1.272-fold) and *EFG1* (1.200-fold) involved in *C. albicans* morphogenesis were found to have no statistical difference compared to the control (*P* > 0.05).

**FIGURE 7:**
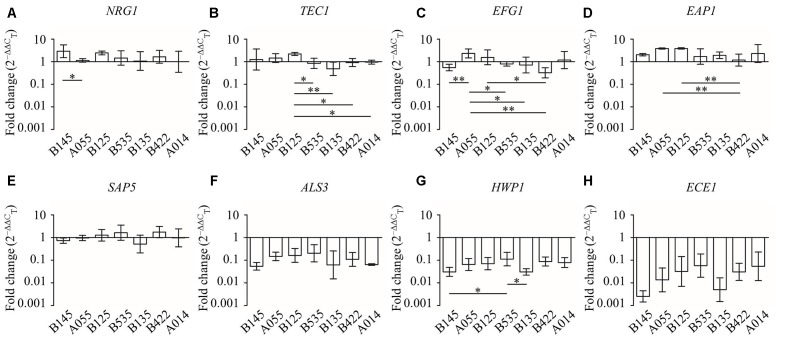
**The expression levels (fold change) of virulence-related genes in *C. albicans* treated with seven different CFS from *L. crispatus*. (A)** *NRG1*, **(B)** *TEC1*, **(C)** *EFG1*, **(D)** *EAP1*, **(E)** *SAP5*, **(F)** *ALS3*, **(G)** *HWP1*, **(H)** *ECE1*. Error bar represents SD from three biological replicates. Comparisons among multiple groups were performed by one-way ANOVA followed by LSD test. ^∗^*P* < 0.05, ^∗∗^*P* < 0.01.

Comparing the different effects of CFS on *C. albicans* gene expression, we found that all seven *L. crispatus* CFS significantly down-regulated the expression of *ALS3*, *HWP,1* and *ECE1* genes (**Figure 6**), and there were no inter-strain difference except for gene *HWP1* (by LSD test: B145 vs. B535, *P* = 0.026; B535 vs. B135, *P* = 0.024) (**Figures 7F–H**). In contrast, the up- and down-regulation phenomena seemed to exist for each of the other five *C. albicans* genes affected by CFS. According to the results of paired comparison, there were statistically significant inter-strain differences for *NRG1*, *TEC1*, *EFG1*, and *EAP1* (**Figures 7A–D**) but not for *SAP5* (**Figure 7E**). Collectively, the results suggested that each *L. crispatus* strains might have slightly different ability and mechanism to regulate *C. albicans* gene expression. Among seven tested *L. crispatus* strains, *L. crispatus* B145 displayed the strongest regulatory activities in terms of down-regulations of *ALS3*, *HWP1*, and *ECE1* expressions, and up-regulation of *NRG1* expression. Interestingly, this was in line with the finding that *L. crispatus* B145 had the strongest activity to inhibit yeast-to-hyphae transition (92.3 ± 9.2% hyphal formation inhibition).

### Characterization of Anti-*Candida*-Related Compounds in CFS Derived from *L. crispatus* B145

In order to get some preliminary information about the chemical nature of the anti-*Candida*-related compounds secreted by *L. crispatus*, the strain B145 was used for further study. This strain was shown to have strong inhibition activities on *C. albicans* growth in the absence and presence of catalase. It also strongly inhibited the hyphal formation and some of the related gene expressions. The susceptibility to heat was evaluated by subjecting the B145 CFS to a number of thermal treatments: 60°C for 30 min; 100°C for 30 min; and 121°C for 15 min. The susceptibility to proteolytic enzymes was investigated by exposing CFS to proteinase-K and trypsin (1 mg/mL; KeyGen Biotech, Nanjing, China) for 3 h at 37°C (Sangmanee and Hongpattarakere, 2014).

**FIGURE 8:**
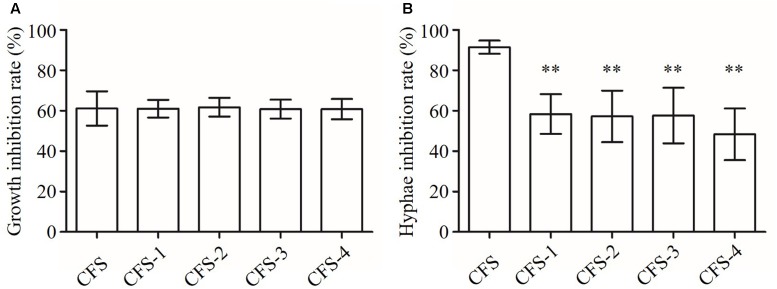
**Inhibition of growth and hyphal formation of *C. albicans* by CFS from *L. crispatus* B145**. CFS: untreated CFS from *L. crispatus* B145, CFS-1: CFS was heated at 80°C for 30 min; CFS-2: CFS was heated at 100°C for 30 min; CFS-3: CFS was heated at 121°C for 15 min; and CFS-4: CFS was treated with proteolytic enzymes. **(A)** Growth inhibition rate (%) = (OD_control_ – OD_CFS_)/OD_control_ × 100, **(B)** Hyphae inhibition rate (%) = (Hyphae%_control_ – Hyphae%_CFS_)/ Hyphae%_control_ × 100. ^∗∗^*P* < 0.01 compared to CFS by Student’s *t*-test.

The B145 CFS had about 60% growth inhibition activity on *C. albicans* after heat and protease treatments with no significant difference compared to that of the untreated CFS (*P* > 0.05) (**Figure 8A**). In addition, the anti-hyphal activities of CFS were significantly reduced after exposing to heat or protease treatments compared to that of the untreated CFS (*P* < 0.01) (**Figure 8B**). However, the residual activity of protease-treated CFS was significantly lower than those of heat treatments (*P* < 0.05).

These primarily results suggested that the growth and the hyphal inhibition activities of *L. crispatus* B145 CFS might be attributed to the antimicrobial compounds with different chemical nature in terms of heat stability and protease susceptibility.

## Discussion

Vulvovaginal candidiasis, primarily caused by *C. albicans*, is one of the most common urogenital diseases affecting women of reproductive-age and associated to the disruption of the healthy vaginal microbiota (Achkar and Fries, 2010). *Lactobacillus*-dominated microbiota is thought to be a valuable biomarker for vaginal health since the *Lactobacillus* species can create a barrier against pathogen invasion (Petrova et al., 2015). *L. crispatus*, *L. gasseri*, and *L. jensenii* were the most frequently isolated species in the vagina of healthy women (Pendharkar et al., 2013; Al et al., 2014; Madhivanan et al., 2015), where *C. albicans* is often a minority co-habitant of vaginal microbiota. In this study, we investigated how these three *Lactobacillus* species affected *C. albicans* growth and yeast-to-hyphae transition, which are implicated in its pathogenesis.

We studied the antimicrobial activities of *Lactobacillus* strains against *C. albicans* using two assays, i.e., the agar overlay method and the microplate-based liquid medium assay. The results were similar in terms of having or not having inhibition activities except for three strains of *L. jensenii* (A083, B021, and B161) (**Table 2** and **Figure 1**). The same phenomenon was also found by other researches. For example, Coman et al. (2014) evaluated the growth inhibition activities of *L. rhamnosus* IMC 501^®^ and *L. paracasei* IMC 502^®^ on different pathogens by several antimicrobial assays, such as agar well diffusion method and liquid co-culture assay. They found that the inhibition results were different within the different methods, and suggested that this might be due to the various antimicrobial mechanisms. In this study, the liquid medium assay using *Lactobacillus* CFS seemed to be more sensitive to assess the antimicrobial activities, where 100% (18/18) of the tested *Lactobacillus* strains showed anti-*Candida* activities rather than 83.3% (15/18) in agar overlay assay. We speculated that the solid agar might block the diffusion of antimicrobial compounds to reach the target *C. albicans*, which might affect those compounds with hydrophobic nature more significantly. This also suggests the necessity of using different methods in assessing the antimicrobial activities and comparing results from different studies in an objective way. Anyhow, our data suggests that the tested *Lactobacillus* strains had moderate to strong anti-*Candida* activities with inter-strain variations. Our results are consistent with some previous studies showing that several strains of lactobacilli were effective at inhibiting the growth of *C. albicans* (Parolin et al., 2015; Hütt et al., 2016).

Lactobacilli are known for their production of various antimicrobial compounds to prevent vaginal infection (Borges et al., 2014). H_2_O_2_ is one of the antimicrobial compounds. We found that *Lactobacillus* strains had different H_2_O_2_ production abilities, ranging from no evidence of H_2_O_2_ production to strong production (**Table 2**). However, all tested *Lactobacillus* strains showed growth inhibition effects on *C. albicans* regardless of H_2_O_2_ production, suggesting that they might produce some antimicrobial compounds in addition to H_2_O_2_. Organic acid production and consequent pH reduction by lactobacilli growth have also been suggested as one of the mechanisms for growth inhibition on *Candida* in previous studies (Jiang et al., 2015). However, we found that the anti-*Candida* activities of the tested CFS might not be largely attributed to low pH (**Figure 1**). Each of CFS from the tested *Lactobacillus* strains showed significantly higher antimicrobial activity than MRS-HCl liquid (pH 4.0) (*P* < 0.01). It suggests that other antimicrobial compounds might play roles in the growth inhibition effects of CFS on *C. albicans*. The antimicrobial compounds, such as bacteriocin-like substances and biosurfactants, have been suggested to contribute to the antagonistic effects of probiotic *Lactobacillus* strains against a variety of vaginal pathogens (Madhu and Prapulla, 2014; Maldonado-Barragán et al., 2016).

In this study, for the first time, the effects of CFS from three species of *Lactobacillus* (*L. crispatus*, *L. gasseri*, and *L. jensenii*) on yeast-to-hyphae transition of *C. albicans* were compared. The ability of yeast-to-hyphae transition is essential for *C. albicans* virulence (Mayer et al., 2013). The hyphae form, which can promote tissue penetration and escape from immune cell, is more prevalent in the infection process than yeast form (Lu et al., 2014). Blocking of virulence traits of pathogens, such as yeast-to-hyphae transition in *C*. *albicans*, has been recently considered as a new antifungal paradigm (Tsang et al., 2012). In this study, *L. crispatus* showed good inhibition activity on hyphal formation (**Figures 4**, **5**). Longitudinal studies have also shown that the presence of *L. crispatus* promoted stability of the vaginal microbiota toward a healthy status (Verstraelen et al., 2009; Santiago et al., 2012). Our findings of *L. crispatus* strains with generally high inhibition activities on *Candida* growth and yeast-to-hyphae transition is very attractive. As far as we know, the clinical approved *L. crispatus* strains are very limited. Our results hint that *L. crispatus* strains are highly worthwhile for further investigation in developing the new antifungal strategies.

Our preliminary results revealed that the antimicrobial compounds from *L. crispatus* B145 against *Candida* growth were heat stable and protease resistance. But the same treatments would greatly reduce the residual activity against yeast-to-hyphae transition. This hints that the growth and yeast-to-hyphae transition inhibition activities would be attributed to different secretory antimicrobial compounds of *L. crispatus* B145. Tendulkar et al. (2007) have reported to identify a heat stable and protease resistance biosurfactant compound, which had anti-fungi activity. The compound was characterized to be lipopeptide in chemical nature. To further study whether *L. crispatus* B145 was able to inhibit different clinical relevant *C. albicans* strains, we tested the growth and hyphae inhibition effect of its CFS on five additional vaginal isolates of *C. albicans*. We found that the growth inhibition rates were from 50.7 to 68.6%, and the hyphae formation inhibition rates were from 77.9 to 91.7% (Supplementary Figure S1). These results confirmed our findings in *C. albicans* ATCC 10231. Further studies to purify the bioactive compounds from *L. crispatus* B145 and to distinguish different compounds in growth and hyphal inhibition would be interesting.

To elucidate the potential mechanisms involved in the inhibition of hyphal formation of *C. albicans* by the *L. crispatus*, we studied the expression levels of five hyphae-specific genes, i.e., *ALS3*, *HWP1*, *ECE1*, *EAP1*, and *SAP5*, in response to *L. crispatus* CFS under hyphae-inducing conditions. These genes encode proteins that are essential for hyphal formation and also play roles in *Candida* pathogenesis. *ALS3* encodes Als3, which is a member of the agglutinin-like sequence gene family and contributes to the invasion of cells and subsequently cell damage (Liu and Filler, 2011). *HWP1* encodes a cell wall mannose protein, which functions as an adhesion, required for hyphal formation and yeast adhesion to epithelial cells (Orsi et al., 2014a). *ECE1* encodes a membrane protein, which is essential for cell elongation and biofilm formation (Fan et al., 2013). *EAP1* gene encodes a glycosylphosphatidylinositol-anchored, glucan-cross-linked cell wall protein, in adhesion and biofilm formation (Li and Palecek, 2003). *SAP5* encodes a member of the secreted aspartic proteases family, which are important for the pathogenesis of candidiasis (Sanglard et al., 1997). The results of qRT-PCR analysis revealed that all seven *L. crispatus* CFS significantly down-regulate the expression of hyphal genes *ALS3*, *HWP1*, and *ECE1*, associated with the inhibition of hyphal formation. Moreover, *C. albicans* hyphal maintenance requires specific transcriptional regulation mechanisms (Fan et al., 2013). We measured the expression of positive regulator genes of *EFG1* and *TEC1*, and the negative regulator gene of *NRG1* (Braun et al., 2001; Argimón et al., 2007; Daniels et al., 2015). Expression of the negative hyphal regulator gene *NRG1* was found to be significantly up-regulated in *C. albicans* in the presence of *L. crispatus* CFS, implying that *L. crispatus* might repress the expression of hyphae-specific genes in a *NRG1*-dependent manner. To the best of our knowledge, this is the first study demonstrating the role of *L. crispatus* in inhibition of *C. albicans* virulence-related gene expressions. Interestingly, *L. crispatus* B145 was shown to strongly up-regulate *NRG1* expression (3.398-fold) and down-regulate *ALS3* (0.057-fold), *HWP1* (0.033-fold), and *ECE1* (0.003-fold) gene expressions. If the antibodies are available, experiments such as Western blot would further confirm this finding.

## Conclusion

The present study demonstrated the *in vitro* inhibitory effects of vaginal *L. crispatus, L. gasseri, and L. jensenii* on *C. albicans* growth and hyphal formation. *L. crispatus* had a generally better ability to inhibit *C. albicans* and might down-regulate the expression of hyphae-specific genes *ALS3*, *HWP1*, and *ECE1* in a *NRG1*-dependent manner. The effects might be due to their productions of some secretory antimicrobial compounds in addition to H_2_O_2_ and organic acids. The growth and hyphal inhibition compounds from *L. crispatus* B145 seemed to be different in chemical nature. Further investigations on *L. crispatus* B145 and other clinical isolates would facilitate the development of probiotic agents with great potentials against *C. albicans*.

## Author Contributions

SW and TL designed experiments, analyzed data and wrote the paper; SW and QW carried out experiments; LY helped with assay set-up; HZ supervised the project and paper writing; EY gave critical review on the manuscript.

## Conflict of Interest Statement

The authors declare that the research was conducted in the absence of any commercial or financial relationships that could be construed as a potential conflict of interest.

## Abbreviations

*ALS3*: agglutinin-like protein 3
CD: colony diameter
CFS: cell-free supernatant
*EAP1*: extracellular adherence protein 1
*ECE1*: extent of cell elongation protein 1
*EFG1*: enhanced filamentous growth 1
FBS: fetal bovine serum
GIA: growth inhibitory activity
*GSP1*: GTP binding protein 1
*HWP1*: hyphal wall protein 1
IZD: inhibition zone diameter
MRS: Man Rogosa and Sharpe
*NRG1*: negative regulator of glucose-repressed 1
OD: optical density
PBS: phosphate buffered saline
qRT-PCR: quantitative real-time reverse transcription PCR
*SAP5*: secreted aspartyl proteinase 5
SD: standard deviation
*TEC1*: transposon enhancement control 1
VVC: vulvovaginal candidiasis
YPD: yeast extract-peptone-dextrose
