# Supplementary material for: Antimicrobial Compounds Produced by Vaginal Lactobacillus crispatus Are Able to Strongly Inhibit Candida albicans Growth, Hyphal Formation and Regulate Virulence-related Gene Expressions
Source: Front Microbiol. 2017 Apr 4;8:564. doi: 10.3389/fmicb.2017.00564 (PMC5378977; doi:10.3389/fmicb.2017.00564)
Supplement: Supplementary file 1 [file Image_1.pdf]

Supplementary Material

Antimicrobial Compounds Produced by Vaginal *Lactobacillus crispatus* Are Able to Strongly Inhibit *Candida albicans* Growth, Hyphal Formation and Regulate Virulence-related Gene Expressions

Shuai Wang, Qiangyi Wang, Ence Yang, Ling Yan, Tong Li*, Hui Zhuang*

*** Correspondence:** Tong Li: tongli08@vip.sina.com, Hui Zhuang: zhuangbmu@126.com

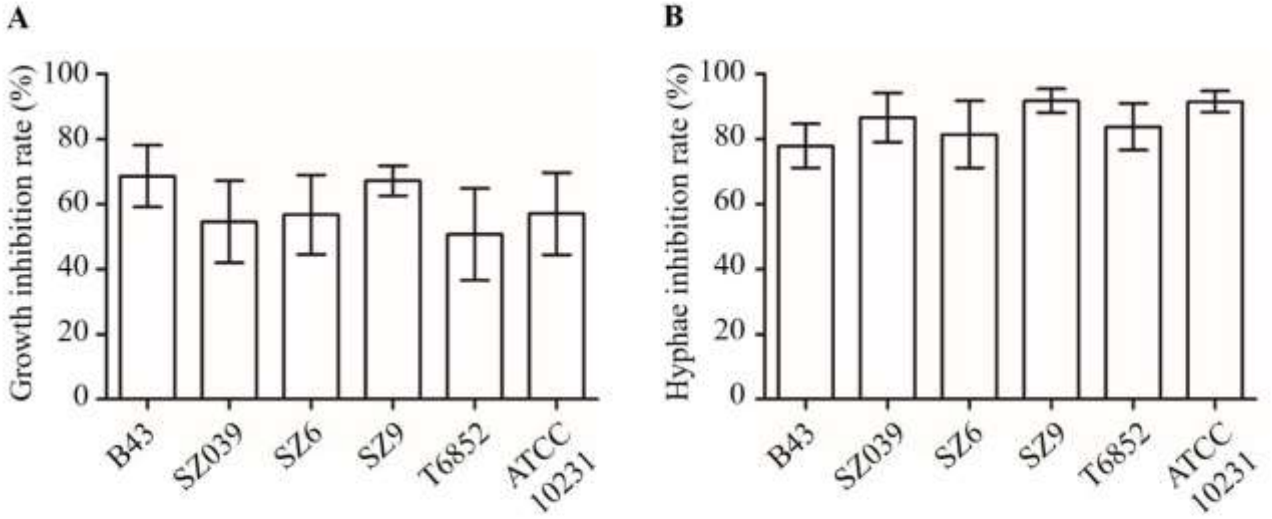

Figure S1. Inhibition of growth and hyphal formation of 6 *C. albicans* isolates by CFS from *L. crispatus* B145. (A) Growth inhibition rate (%), (B) Hyphae inhibition rate (%). *C. albicans* B43, SZ039, SZ6, SZ9, and T6852 were vaginal isolates. Growth inhibition rate (%) = $(OD_{control} - OD_{CFS}) / OD_{control} \times 100$, Hyphae inhibition rate (%) = $(Hyphae\%_{control} - Hyphae\%_{CFS}) / Hyphae\%_{control} \times 100$.
